# Tung Oil Thermal Treatment Improves the Visual Effects of Moso Bamboo Materials

**DOI:** 10.3390/polym14061250

**Published:** 2022-03-20

**Authors:** Tong Tang, Benhua Fei, Wei Song, Na Su, Fengbo Sun

**Affiliations:** 1 School of Art & Design, Qilu University of Technology (Shandong Academy of Sciences), Jinan 250353, China; 2 Key Laboratory of Bamboo and Rattan Science and Technology of the State Forestry Administration, Department of Biomaterials, International Center for Bamboo and Rattan, Beijing 100102, China; feibenhua@icbr.ac.cn (B.F.); j5international@163.com (W.S.); yuhesu122216@126.com (N.S.)

**Keywords:** tung oil, bamboo, thermal treatment, color

## Abstract

Color is one of the most important characteristics of a material’s appearance, which affects the additional value of bamboo and psychological feelings of users. Previous studies have shown that the dimensional stability, mildew resistance and durability of bamboo were improved after tung oil thermal treatment. In this study, the effects of tung oil thermal treatment on bamboo color at different temperatures and durations of time were investigated. The results show that the lightness (L*) of bamboo decreased as the tung oil temperature or duration of time increased. The red–green coordinates (a*) and color saturation (C*) of bamboo were gradually increased as the tung oil temperature rose from 23 °C to 160 °C, while the a* and C* were gradually decreased when the temperature continued to rise from 160 °C to 200 °C. There was no significant difference in the yellow–blue coordinates (b*) of bamboo when the duration was prolonged from 0.5 h to 3 h with tung oil thermal treatment at 140 °C. Eye movement data show that the popularity of bamboo furniture was significantly improved at 23–100 °C and slightly improved at 160–180 °C with tung oil treatment. Therefore, tung oil thermal treatment plays a positive role in improving visual effects and additional value of bamboo.

## 1. Introduction

Bamboo is an excellent biomaterial that improves the living environments of humans and has lightweight, good mechanical properties, processes properties and visual properties [1,2]. Bamboo has been widely used in architecture, decoration and furniture. Color is an important characteristic for the evaluation of the application value of bamboo [3,4]. The color of bamboo is perceived by human eyes at a light wavelength at 400–700 nm after absorption and reflection on the bamboo surface, relating to the color system composed of chromophores and auxochromes. However, bamboo is prone to deformation and mildew. Therefore, it is necessary to modify bamboo before it is used as an engineering material.

In order to improve the durability of bamboo, the properties of bamboo can be improved by mechanical compression, surface coating, thermal treatment, in situ polymerization and so on [5,6,7,8,9]. Oil thermal treatment is considered to be an effective industrial modification method for improving the dimensional stability, mildew resistance and durability of bamboo and wood [10,11,12]. Compared with thermal treatment in steam [13,14] or a nitrogen atmosphere [15], oil thermal treatment can protect wood or bamboo from mold and fungi decay as well as prevent moisture access to its cell walls in the long term, as oil is not only a heat transfer medium but also an excellent modifier. Oil had been used as protective surface coating for over thousands years. Additionally, oil treatment not only improves wood properties but also enhances the aesthetic of wood. In addition, tung oil, as a vegetable oil, is friendly for the environment and wood itself [16]. The effect of oil thermal treatment on wood color is mainly related to the changes in chromophores and auxochromes in lignin, extracts and hemicellulose [17,18]. The oxidative polymerization of oil influences the color of the material, and the more oil absorbed by the material, the deeper the color of the material. For example, after Dubey et al. thermally treated radiata pine with linseed oil at 180 °C, the lightness (L*) of the radiata pine significantly decreased, whereas the red–green coordinates (a*) and yellow–blue coordinates (b*) increased, which may be related to the oil absorption of radiata pine and the move of pyrolysis products, including quinine, low-molecular-weight sugar and amino acid to the surface of radiata pine [19].

Previous studies have shown that the synergistic effect of tung oil and thermal treatment (100–200 °C) could improve the dimensional stability and mildew resistance of bamboo and maintain the excellent mechanical properties of bamboo [12,20]. Additionally, tung oil can separate oxygen from bamboo during the treatment process, with less degradation of the bamboo’s chemical structure compared with thermal treatment in air, allowing the excellent mechanical properties of bamboo to be maintained [17]. Tung oil is a transparent oil with an orange color containing a mass of unsaturated conjugated groups compared with other natural vegetable oils, such as soybean oil and flaxseed oil; thus, the natural drying time of tung oil is significantly shorter than that of the other oils. For instance, the natural drying time of tung oil is approximately one-tenth of that of linseed oil [21,22,23]. Moreover, tung oil is an excellent water repellent, even after severe aging and weathering [24]. Therefore, tung oil is more advantageous in industrial applications. At present, no systematic study on the effect of tung oil thermal treatment on bamboo color has been carried out. Moreover, due to unique chemical property of tung oil, the effects and mechanisms of thermal treatment with tung oil on bamboo could be different from other oils. In view of the importance of bamboo color for its application in the living environment, this study examined the effect of different temperatures (23–200 °C) and durations (0.5–3 h) of tung oil thermal treatment on bamboo color. The visual–physical and visual–psychological data were measured and analyzed to provide a theoretical basis for the application of tung oil in bamboo modification.

## 2. Materials and Methods

### 2.1. Materials

**Figure 1:**
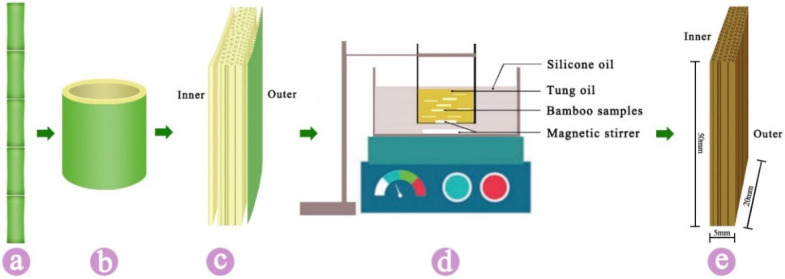
Schematic presentation of sample preparation. (**a**) Moso bamboo, (**b**) bamboo culm, (**c**) bamboo sample, (**d**) bamboo thermal treatment with tung oil and (**e**) bamboo sample.

Five-year-old moso bamboo (*Phyllostachys edulis* (Carr.)J. Houz) was obtained from Xuancheng, Anhui, China. Moso bamboo from 1.5 m (height from base) to 3.5 m in height was used in this study. Defect-free bamboo materials were dried at room temperature and cut from the center region to dimensions of 20 × 50 × 5 mm^3^ (longitudinal × tangential × radial), as shown in Figure 1a–c. Samples were then kept in a climate-controlled room until the moisture content reached approximately 12% before use. Tung oil was purchased from Emperor’s craftsman, Shanghai, China.

### 2.2. Sample Preparation

Moso bamboo samples were treated with tung oil at 23 °C, 100 °C, 120 °C, 140 °C, 160 °C, 180 °C and 200 °C for 3 h (Figure 1d). Furthermore, in order to analyze the color as influenced by the duration of treatment, the bamboo samples were thermal treated with tung oil at 140 °C for 0.5 h, 1 h and 3 h. The 140 °C variable was chosen for a further analysis of how color is influenced by treatment duration, mainly because of the better mechanical performance at 140 °C than other temperatures (range from 23 to 200 °C) [12,20]. During thermal treatment of samples, the treatment temperature was maintained constantly within ±2 °C. After tung oil thermal treatment, the excess oil on samples’ surface was wiped off, and the samples were then naturally dried. Following our conventions, 140 °C–3 h is the abbreviation of bamboo that underwent tung thermal treatment at 140 °C for 3 h, and likewise for the other treatments.

### 2.3. Visual–Physical Quantification

The color of bamboo before and after thermal treatment with tung oil was measured using a colorimeter (CC-6834, BYK-Gardner, Grazrid, Germany), referring to color space CIE L*a*b*. Measurements were carried out by a D65 illuminant and 10° standard observer. To reduce errors, color parameters were measured at the center position of the sample surface. Furthermore, the measurement was performed more than 20 times to verify the results.

The CIE L*a*b* is one of the most frequently used methods to quantify surface color, as it describes the color as human eyes would recognize it. The relative color changes ΔL*, Δa* and Δb* were calculated by L*, a* and b* before and after the same sample was treated (for example: Δa* = a*_after treated_ − a*_before treated_).

From the relative color changes ΔL*, Δa* and Δb*, the total color change ΔE* was calculated by Equation (1):

(1) $$\Delta E*=\sqrt{{\Delta a*}^{2}+{\Delta b*}^{2}+{\Delta L*}^{2}}\;$$

Based on the L*, a* and b* color coordinates, the color saturation (C*) was calculated according to Equation (2):

(2) $$C*=\sqrt{{a*}^{2}+{b*}^{2}}\;$$

The statistical analysis of color parameters was carried out with the use of IBM SPSS Statistics software and based on the least significant difference (LSD) or one-way ANOVA, with a significance level (p) of 0.05. The same superscript letters (a, b, c, d, e, f or g) marked in the same column indicate there were no significant differences between them.

### 2.4. Visual–Psychological Quanification

The effect of bamboo color changes on the psychological feelings of users was quantified using an eye tracker (Tobii X120, Tobii Technology AB, Danderyd, Sweden). A total of 40 participants were randomly selected from college students at Qilu University of Technology with normal vision, aged between 18 and 26 years old. Three different types of furniture, including two different styles of tables and one cabinet, were designed. The bamboo furniture models with corresponding colors after different tung oil treatments were created using graphic design software, which was used to quantify the changes in users’ visual psychology. In the eye movement experiment, the same type of bamboo furniture that had undergone different tung oil treatments with varying temperature or treatment duration was put in a picture, and twelve groups of tests were designed in total. After the participants entered a laboratory with sound insulation and uniform light, a pre-experiment was conducted so participants could familiarize themselves with the testing process. Subsequently, the formal experiment began after the participants relaxed their eyes for one minute. The experiments involved observing bamboo furniture and making a choice about their favorite piece of furniture. To quantify the visual psychology of different types tung-oil-treated bamboo furniture, the eye tracker automatically recorded the eye fixation duration, fixation count and a hot-spot map of the participants as they observed different colors and different types of furniture during the experiment. The total fixation duration and fixation count of each participant was different, so it is more accurate to express them as a percentage.

## 3. Results and Discussion

The visual-physical quantity depends on color tone, lightness and saturation. The color tone is determined by the dominant wavelength of light reflected from the surface of the material. In the visible spectrum, different wavelengths of light cause different visual effects, resulting in red, orange, yellow, green, blue, purple or other corresponding tones. Lightness is the degree of color brightness, which refers to the reflection coefficient of light and depends on the intensity of the light. Saturation refers to the purity of color and depends on the range of the wavelength of light reflected from the surface.

Bamboo is usually processed into standard engineering materials, such as laminated timber and flattened timber after removing bamboo bark. Due to the differential radial gradient structure of bamboo, bamboo shows different colors in different radial gradient directions affected by the changes in chemical composition [25]. In this study, the color (tone, lightness and saturation) of bamboo (near the outer layer and near the inner layer) after different tung oil treatments was investigated in detail.

### 3.1. The Effect of Changes in Tung Oil Temperature on Bamboo Color

The color of bamboo after tung oil treatment for 3 h at different temperatures is shown in Table 1. The L* of bamboo near the outer layer was decreased after tung oil treatment, which was decreased from 81.67 (untreated bamboo) to 78.41 (23 °C–3 h). The L* of bamboo near the outer layer continued to decline as tung oil temperature increased, reaching only 37.77 after tung oil treatment at 200 °C. The a* of bamboo after tung oil treatment was higher than that of the untreated bamboo, indicating that the redness of the bamboo increased after tung oil treatment. When the tung oil temperature increased from 23 °C to 160 °C, the a* of bamboo near the outer layer gradually increased from 12.64 to 18.12, while the a* of bamboo near the outer layer decreased to 14.94 as the tung oil temperature increased to 200 °C. No significant difference in the b* of bamboo after the increase in tung oil temperature from 23 °C to 160 °C was observed, but the b* within this temperature range was significantly higher than that of the bamboo that had undergone an increase in tung oil temperature from 180 °C to 200 °C. The variation trend of C* of bamboo was similar to that of a*, and the only difference was that the C* of bamboo that had undergone tung oil thermal treatment at 200 °C was lower than that of untreated bamboo.

The L* of untreated bamboo from near the outer layer was lower than that from near the inner layer, whereas the a* and b* were higher than that from near the inner layer. The bamboo color is mainly related to the chemical structure of lignin and extractives [26,27]. Wei et al. measured the chemical composition of inner, middle and outer moso bamboo layers, which showed that the content of lignin in the outer layer was higher than in the inner layer [28]. The content of lignin in the outer layer was higher than in the inner layer of bamboo, which indicates a high bulk density increases the chromophore concentration in the outer layer, leading to more chromophores in the outer layer [29]. Hence, the color intensity of the bamboo’s outer layer was increased, and a reduction in lightness could be observed. Although bamboo has a gradient structure, the color change rule of bamboo near the inner layer was similar to that of the outer layer (Table 1).

**Figure 2:**
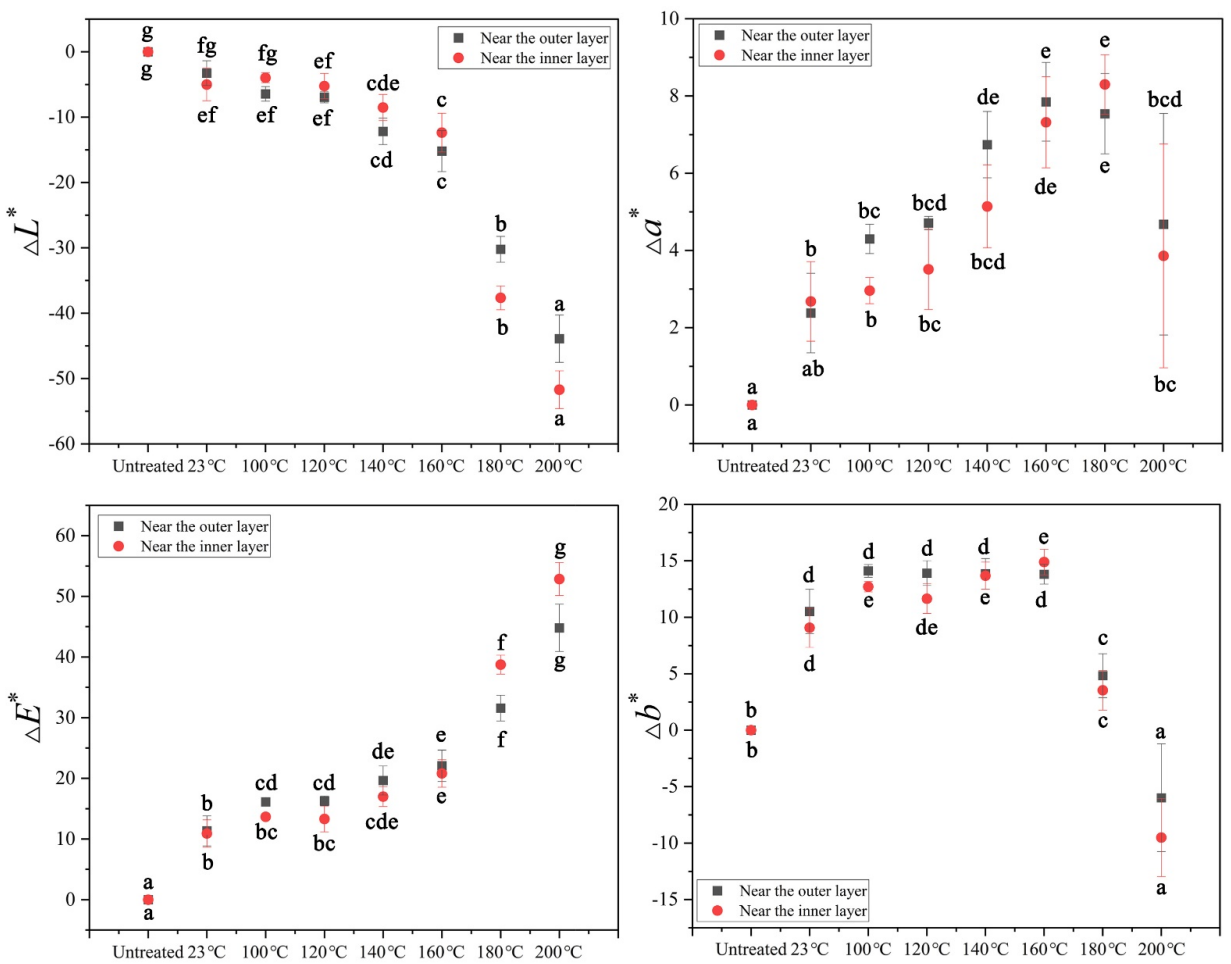
The variation in bamboo color after tung oil treatment at different temperatures. (* Note: Mean values followed by the same superscript letters (a, b, c, d, e, f or g) in the same column are not significantly different at <0.05. Values in parentheses are standard deviations. Outer: the surface near the outer layer of bamboo. Inner: the surface near the inner layer of bamboo.).

In order to further analyze the effect of tung oil temperature on bamboo color, statistical analysis was conducted on the changes in color, as shown in Figure 2. Both tung oil thermal treatment and tung oil treatment at room temperature changed bamboo color, and the ΔE* increased with rising tung oil temperature. A significant color difference was observed between 23 °C–3 h and untreated bamboo, which was mainly related to the oxidation of tung oil on bamboo. Yoo et al. reported that the L* of wood was decreased, whereas the a* and the b* were increased on the tung-oil-finished wood surface [22], which shows similar results with tung-oil-treated bamboo at 23 °C. The a* and b* of bamboo were gradually decreased as the tung oil treatment temperature increased from 160 °C to 200 °C. The same trend was also found in wood after thermal treatment [19,30,31,32].

With rising tung oil temperature, the ΔE* was gradually increased (Table S1), which was closely related to the degradation of bamboo chemical components and the aging of tung oil on bamboo. The chemical structure of lignin and extractives changed after thermal treatment, resulting in the variation in bamboo color [33]. Lignin is an aromatic compound that produces a significant number of chromophores during thermal treatment [34]. Previous studies have suggested that the β-O-4 bond in the propyl structure of the benzene ring side chain is acidified and broken during thermal treatment, which reduces the esterification structure and increases the content of phenolic hydroxyl [35,36,37,38]. The phenolic hydroxyl is gradually oxidized to quinone compounds, leading to the increase in a* and b*. In addition, the dehydration reaction occurs in conjugated C=C in the aromatic ring and C=O during thermal treatment, resulting in the increase in ketones and conjugated carbonyl groups in lignin [39]. The increase in conjugated structure and extension of conjugated system leads to increased L* [27,40,41,42]. Extractives mainly include phenolic, alcohol and aldehyde compounds [43], and they can be acid-catalyzed by acetic acid during thermal treatment, which causes the self-condensation and oxidation of compounds, forming a new chromogenic system composed of conjugated double bonds, carbonyl or a quinone structure. The spectral absorption is enhanced and extended to the range of visible light, resulting in the bamboo becoming darker and redder.

Tung oil has major components of α-eleostearic acid (77–82%) with three conjugated double bonds (at carbons 9 *cis*,11 *trans*, and 13 *trans*), oleic acid (3.5–12.7%) with one double bond, and linoleic acid (8–10%) with two nonconjugated double bonds [22]. Partially conjugated double bonds are altered to nonconjugated double bonds after thermal treatment by oxidation, thermal rearrangement or both [44]. Similarly to other vegetable oils [45], the color of tung oil deepens with thermal treatment. The changed chemical structure of tung oil also influences bamboo color. In addition, some studies have demonstrated that more oil absorption results in a deeper color of the material [20,46]. After the thermal treatment and drying processes, an oil layer is formed on the bamboo surface, enhancing surface color changes in bamboo [6]. The chemical structure of bamboo and tung oil change during thermal treatment, and oil absorption changes along with the thermal temperature [12], which comprehensively influence bamboo color.

### 3.2. The Effect of Changes in Tung Oil Treatment Duration on Bamboo Color

The color of bamboo after tung oil thermal treatment at 140 °C under different treatment durations of time is shown in Table 2. With the extension of tung oil treatment duration, the L* of bamboo decreased and the a* of bamboo increased, but the tung oil treatment duration had no significant impact on the b* of bamboo. After thermal treatment with tung oil for over 1 h, the C* of bamboo did not change significantly. Comparing Table 1 with Table 2, the ΔE* of 140 °C–0.5 h was higher than that of 23 °C–3 h but lower than that of 100 °C–3 h. Furthermore, the ΔE* of 140 °C–1 h was higher than that of 120 °C–3 h but lower than that of 140 °C–3 h. Significant differences among the color of bamboo were observed after thermal treatment for 0–1 h at 140 °C. However, the differences reduced with increasing treatment duration.

**Figure 3:**
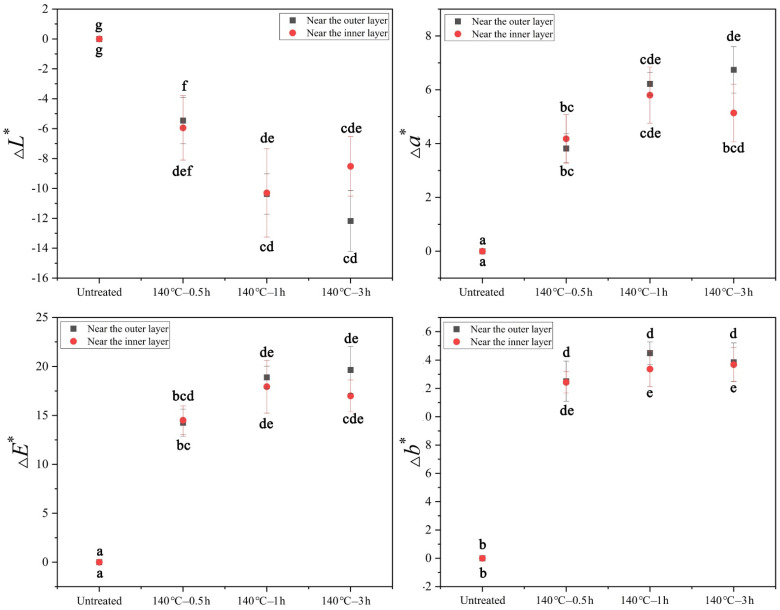
The variation in bamboo color after tung oil treatment at 140 °C for 0–3 h. (* Note: Mean values followed by the same superscript letters (a, b, c, d, e, f or g) in the same column are not significantly different at <0.05. Values in parentheses are standard deviations. Outer: the surface near the outer layer of bamboo. Inner: the surface near the inner layer of bamboo.).

The changes in bamboo color after different tung oil treatment durations are shown in Figure 3. The L* and a* of bamboo significantly changed after tung oil thermal treatment at 140 °C within 0–3 h, which was mainly related to the gradual increase in quinone compounds, ketones and the conjugated system during thermal treatment. The b* of bamboo had no significant changes after tung oil thermal treatment at 140 °C within 0.5–3 h (Figure 3), which indicates a rapid increase in b* occurred in the early thermal treatment and an extended treatment duration had little impact on b*.

The ΔE* was affected by thermal treatment conditions (Table S2) and the type of material. The ΔE* was gradually increased with the thermal temperature rising from 23 °C to 200 °C (Figure 2), which was also gradually increased over the thermal treatment duration, which was extended to 1 h at 140 °C (Figure 3). The ΔE* had greater values after the oil thermal treatment than after air thermal treatment and nitrogen thermal treatment under the same conditions [6]. The ΔE* was also related to the type of material, for example, moso bamboo had an obviously higher value of ΔE* compared with Scots pine after thermally treated in air under the same conditions [6,47]. Nevertheless, bamboo scrimber thermally treated with methyl silicone oil had a similar value of ΔE* to moso bamboo thermally treated with tung oil [11].

### 3.3. The Effect of Tung Oil Treatment on Visual–Psychological Quantification

Eye trackers are used to precisely record the trajectory of human eye movement. Visual responses and characteristics can be obtained from eye movement data. The fixation points of observers are usually distributed across the most important, comprehensible or informative objects. The regions recognized as the interesting spots experience more fixation counts and a longer fixation duration [48,49,50,51]. The hot-spot map as a common representation of fixation duration that appears in the studies of eye movements, which reflects the attention of participants [52,53]. Together, the fixation count, fixation duration and hot-spot map were used to analyze the popularity of different bamboo furniture pieces after tung oil treatment.

**Figure 4:**
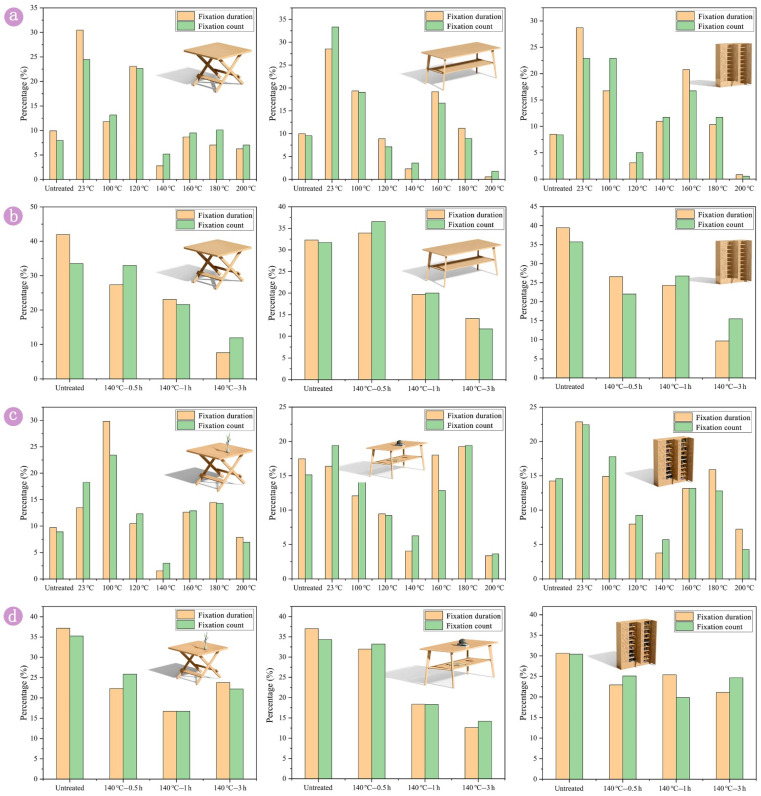
The fix duration and fix count of different bamboo furniture. (**a**,**b**) Furniture was made of the outer layer of bamboo, (**c**,**d**) furniture was made of the inner layer of bamboo.

To simulate bamboo used in the living environment, three different types of furniture were designed and matched to bamboo color after tung oil treatment. The eye movement data show that furniture was made of bamboo after tung oil treatment at 23 °C–3 h, 100 °C–3 h, 160 °C–3 h and 180 °C–3 h experience more fixation counts and a longer fixation duration compared with untreated bamboo (Figure 4), indicating the popularity of bamboo furniture was improved after tung oil treatment at 23 °C, 100 °C, 160 °C and 180 °C. The popularity of bamboo furniture was significantly improved after tung oil treatment at 23 °C and 100 °C, whereas a slightly improved popularity of bamboo furniture was observed after tung oil treatment at 160 °C and 180 °C (Figure 4). The popularity of bamboo furniture after tung oil thermal treated at 140 °C and 200 °C decreased according to the eye movement data. A longer fixation duration and greater fixation count of bamboo furniture made with untreated bamboo compared with that furniture made of bamboo treated at 140 °C–0.5 h, 140 °C–1 h and 140 °C–3 h (Figure 4b,d), which suggests the participants had a greater preference for untreated bamboo compared to the bamboo treated with tung oil at 140 °C.

**Figure 5:**
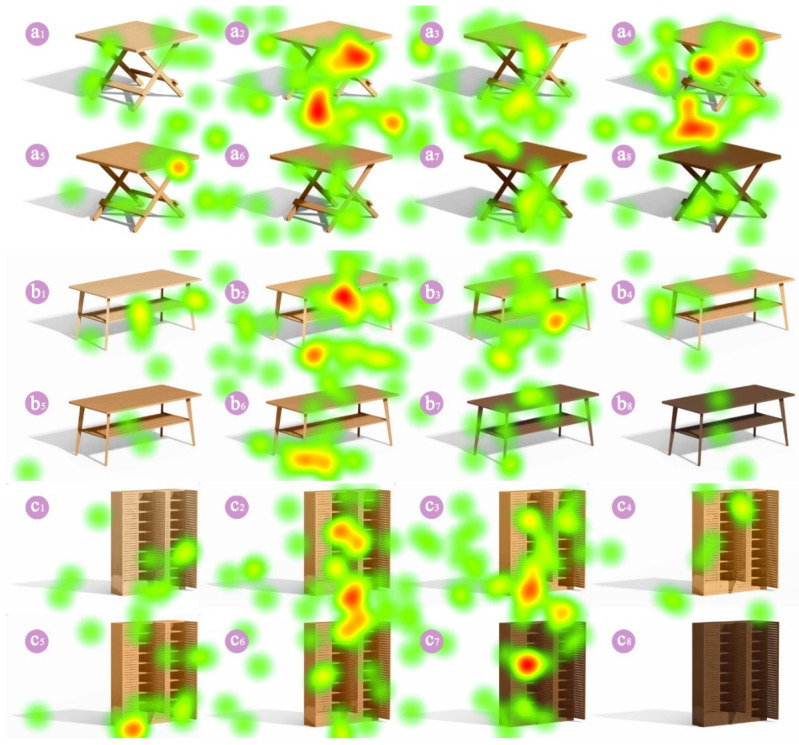
The hot-spot map of different types of bamboo furniture. Bamboo (near the outer layer) after thermal treatment with tung oil for 3h: (**a_1_**–**c_1_**) untreated, (**a_2_**–**c_2_**) 23 °C, (**a_3_**–**c_3_**) 100 °C, (**a_4_**–**c_4_**) 120 °C, (**a_5_**–**c_5_**) 140 °C, (**a_6_**–**c_6_**) 160 °C, (**a_7_**–**c_7_**) 180 °C and (**a_8_**–**c_8_**) 200 °C.

The fix duration and fix count had some differences among the different types of furniture, and the hot-spot map for bamboo furniture is shown in Figure 5. In the hot-spot map, the area of the furniture was overlaid with red and yellow colors, indicating that this area received a longer total fixation duration. By contrast, the green color indicates a shorter fixation duration. The bamboo treated at 23 °C–3 h, 100 °C–3 h, 160 °C–3 h and 180 °C–3 h had a larger area of the red and yellow colors, which suggests that the participants had a greater preference for bamboo that had been treated at 23 °C–3 h, 100 °C–3 h, 160 °C–3 h and 180 °C–3 h. Interestingly, the area of the red and yellow colors for bamboo treated at 120 °C–3 h and 200 °C–3 h was gradually decreased as furniture volume increase. According to the hot-spot map, the table (small furniture) that had been treated with tung oil at 200 °C was more popular than the cabinet (large furniture).

Previous studies have shown that tung oil thermal treatment could improve the dimensional stability and mildew resistance of bamboo, and the durability of bamboo was gradually improved with the increase in tung oil thermal temperature [12]. According to a comprehensive analysis of bamboo properties and the popularity of bamboo furniture, the popularity of bamboo after tung oil thermal treatment at 23 °C and 100 °C was significantly improved and the durability was slightly improved, which may be more suitable for furniture or interior decorative materials with high appearance requirements. By contrast, the popularity of bamboo after tung oil thermal treatment at 160 °C and 180 °C was slightly improved and the durability was significantly improved, which is more suitable for furniture or engineering materials with high durability requirements. Although the durability of bamboo after tung oil thermal treatment at 200 °C had the greatest improvement, the popularity of bamboo was relatively low, suggesting that bamboo treated with tung oil at 200 °C may be more applicable to outdoor engineering materials, such as floorings and fencing or small-area decoration materials. Thus, the application of tung oil modification technology to bamboo not only improved the durability but also improved the visual effects of bamboo.

## 4. Conclusions

The oil absorption of bamboo and chemical structure of bamboo and tung oil comprehensively influenced bamboo color. Bamboo became darker with the increase in tung oil treatment temperature and duration of tung oil treatment time. The L* of bamboo after tung oil thermal treatment at 200 °C was decreased by more than 50% compared with untreated bamboo. Bamboo became redder and had a higher saturation as the tung oil temperature rose from 23 °C to 160 °C. When the tung oil treatment temperature was higher than 180 °C, bamboo became greener and had a lower saturation with increasing tung oil temperature. The L* and a* of bamboo were closely related to tung oil treatment duration. However, the b* of bamboo had no significant change over the duration of tung oil treatment from 0.5 h to 3 h at 140 °C. According to eye movement data, the popularity of furniture made by bamboo treated at 23 °C–3 h, 100 °C–3 h, 160 °C–3 h and 180 °C–3 h was increased compared with that made with untreated bamboo. Comprehensive analysis of bamboo properties and the popularity of bamboo furniture suggests that bamboo after tung oil thermal treatment at 23 °C and 100 °C is more suitable for engineering materials with high appearance requirements, whereas bamboo after tung oil thermal treatment at 160 °C and 180 °C is more suited to engineering materials with high durability requirements. The application of tung oil modification technology in the production of bamboo engineering materials not only improved the durability of bamboo but also improved the visual effects of bamboo, which would expand the application field of bamboo.

## Figures and Tables

**Table 1:** The color of bamboo after tung oil treatment at different temperatures.

Treatment	Lightness L*		Red–Green Coordinates a*		Yellow–Blue Coordinates b*		Color Saturation C*	
	Outer	Inner	Outer	Inner	Outer	Inner	Outer	Inner
Untreated	81.67 ^g^	84.11 ^g^	10.26 ^a^	8.75 ^a^	25.56 ^b^	23.63 ^b^	27.55 ^a^	25.20 ^b^
	(0.80)	(0.66)	(0.33)	(0.40)	(0.68)	(0.69)	(0.73)	(0.75)
23 °C–3 h	78.41 ^fg^	79.10 ^ef^	12.64 ^ab^	11.43 ^b^	36.08 ^d^	32.71 ^d^	38.23 ^bc^	34.66 ^cd^
	(1.90)	(2.50)	(1.03)	(1.03)	(1.96)	(1.74)	(2.19)	(1.91)
100 °C–3 h	75.23 ^ef^	80.14 ^fg^	14.56 ^bc^	11.71 ^b^	39.67 ^d^	36.34 ^e^	42.26 ^cd^	38.18 ^def^
	(1.11)	(0.76)	(0.38)	(0.34)	(0.56)	(0.46)	(0.55)	(0.46)
120 °C–3 h	74.72 ^ef^	78.87 ^ef^	14.97 ^bcd^	12.26 ^bc^	39.47 ^d^	35.28 ^de^	42.22 ^cd^	37.35 ^de^
	(0.84)	(1.93)	(0.17)	(1.04)	(1.10)	(1.31)	(1.05)	(1.56)
140 °C–3 h	69.49 ^cd^	75.59 ^cde^	17.01 ^de^	13.89 ^bcd^	39.41 ^d^	37.31 ^e^	42.92 ^d^	39.82 ^ef^
	(2.03)	(1.99)	(0.86)	(1.07)	(1.35)	(1.20)	(1.57)	(1.22)
160 °C–3 h	66.47 ^c^	71.75 ^c^	18.12 ^e^	16.07 ^de^	39.37 ^d^	38.53 ^e^	43.35 ^d^	41.76 ^f^
	(3.13)	(2.95)	(1.02)	(1.18)	(0.87)	(1.13)	(0.94)	(1.30)
180 °C–3 h	51.46 ^b^	46.47 ^b^	17.80 ^e^	17.05 ^e^	30.39 ^c^	27.18 ^b^	35.22 ^b^	32.09 ^c^
	(1.98)	(1.82)	(1.04)	(0.77)	(1.94)	(1.76)	(2.11)	(1.88)
200 °C–3 h	37.77 ^a^	32.39 ^a^	14.94 ^bcd^	12.60 ^bc^	19.58 ^a^	14.12 ^a^	24.65 ^a^	18.94 ^a^
	(3.63)	(2.39)	(2.87)	(2.90)	(4.77)	(3.45)	(5.45)	(4.42)

* Note: Mean values followed by the same superscript letters (a, b, c, d, e, f or g) in the same column are not significantly different at <0.05. Values in parentheses are standard deviations. Outer: the surface near the outer layer of bamboo. Inner: the surface near the inner layer of bamboo.

**Table 2:** The color of bamboo after tung oil treatment at different durations of time.

Treatment	Lightness L*		Red–Green Coordinates a*		Yellow–Blue Coordinates b*		Color Saturation C*	
	Outer	Inner	Outer	Inner	Outer	Inner	Outer	Inner
Untreated	81.67 ^g^	84.11 ^g^	10.26 ^a^	8.75 ^a^	25.56 ^b^	23.63 ^b^	27.55 ^a^	25.20 ^b^
	(0.80)	(0.66)	(0.33)	(0.40)	(0.68)	(0.69)	(0.73)	(0.75)
140 °C–0.5 h	76.21 ^f^	78.15 ^def^	14.09 ^bc^	12.92 ^bc^	38.07 ^d^	36.06 ^de^	40.60 ^cd^	38.31 ^def^
	(1.54)	(2.16)	(0.55)	(0.89)	(1.42)	(0.76)	(1.46)	(0.88)
140 °C–1 h	71.31 ^de^	73.81 ^cd^	16.48 ^cde^	14.55 ^cde^	40.05 ^d^	37.01 ^e^	43.31 ^d^	39.77 ^ef^
	(1.35)	(2.96)	(0.43)	(1.04)	(0.78)	(1.23)	(0.83)	(1.44)
140 °C–3 h	69.49 ^cd^	75.59 ^cde^	17.01 ^de^	13.89 ^bcd^	39.41 ^d^	37.31 ^e^	42.92 ^d^	39.82 ^ef^
	(2.03)	(1.99)	(0.86)	(1.07)	(1.35)	(1.20)	(1.57)	(1.22)

* Note: Mean values followed by the same superscript letters (a, b, c, d, e, f or g) in the same column are not significantly different at <0.05. Values in parentheses are standard deviations. Outer: the surface near the outer layer of bamboo. Inner: the surface near the inner layer of bamboo.

## Data Availability

All data or used during the study appear in the submitted article.

## Supplementary Materials

The following supporting information can be downloaded at: https://www.mdpi.com/article/10.3390/polym14061250/s1, Table S1: Visualizations of bamboo after tung oil treatment at different temperature, Table S2: Visualizations of bamboo after tung oil treatment at different duration of time.
