# Supplementary material for: Tung Oil Thermal Treatment Improves the Visual Effects of Moso Bamboo Materials
Source: Polymers (Basel). 2022 Mar 20;14(6):1250. doi: 10.3390/polym14061250 (PMC8955884; doi:10.3390/polym14061250)
Supplement: Supplementary file 1 [file polymers-14-01250-s001.zip › polymers-1635695-supplementary.pdf]

Supplementary material

Tung oil thermal treatment improves the visual effects of moso bamboo materials

Tong Tang [1,2,*], Benhua Fei [2], Wei Song [2], Na Su [2], Fengbo Sun [2,*]

[1] School of Art & Design, Qilu University of Technology (Shandong Academy of Sciences), Jinan 250353, China
[2] Key Laboratory of Bamboo and Rattan Science and Technology of the State Forestry Administration, Department of Biomaterials, International Center for Bamboo and Rattan, Beijing 100102, China; feibenhua@icbr.ac.cn (B.F.); j5international@163.com (W.S.); yuhesu122216@126.com (N.S.)
* Correspondence: tangtong@qlu.edu.cn (T.T.); sunfengbo@icbr.ac.cn (F.S.)

Table S1. Visualizations of bamboo after tung oil treatment at different temperature.

**Treatment**	**Visual**
Untreated	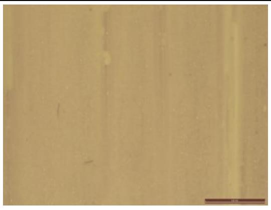
23 °C-3 h	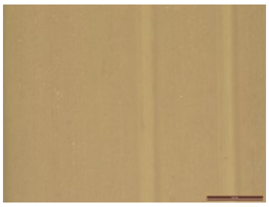
100 °C-3 h	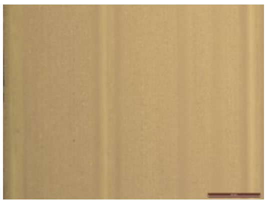
120 °C-3 h	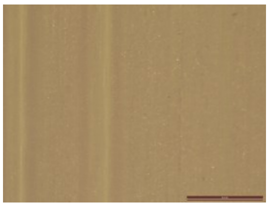
140 °C-3 h	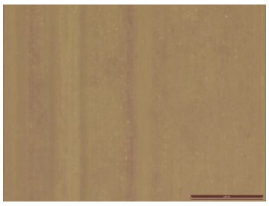
160 °C-3 h	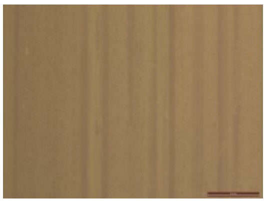
180 °C-3 h	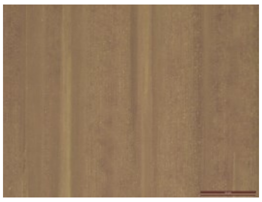
200 °C-3 h	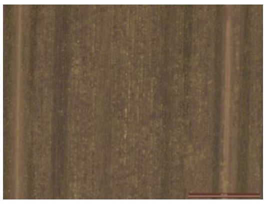

Table S2. Visualizations of bamboo after tung oil treatment at different duration of time.

Treatment	Visual
Untreated	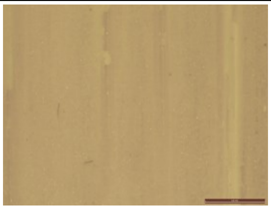
140 °C-0.5 h	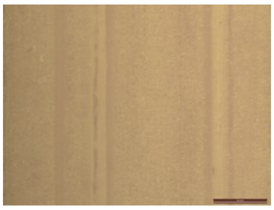
140 °C-1 h	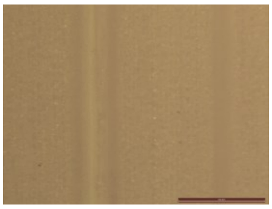
140 °C-3 h	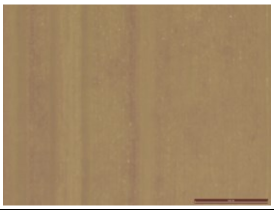
